# Uncovering novel genes for drought stress in rice at germination stage using genome wide association study

**DOI:** 10.3389/fpls.2024.1421267

**Published:** 2024-08-01

**Authors:** Mvuyeni Nyasulu, Qi Zhong, Xiansheng Li, Xu Liu, Zhengjie Wang, Liang Chen, Haohua He, Jianmin Bian

**Affiliations:** ^1^ Key Laboratory of Crop Physiology, Ecology, and Genetic Breeding, Ministry of Education, Jiangxi Agricultural University, Nanchang, China; ^2^ College of Agronomy, Jiangxi Agricultural University, Nanchang, China

**Keywords:** rice, drought tolerance, genome-wide association study (GWAS), QTLs, genetic diversity

## Abstract

**Introduction:**

Breeding rice with drought tolerance for harsh environments is crucial for agricultural sustainability. Understanding the genetic underpinnings of drought tolerance is vital for developing resilient rice varieties. Genome-wide association studies (GWAS) have emerged as pivotal tools in unravelling the complex genetic architecture of traits like drought tolerance, capitalizing on the natural genetic diversity within rice germplasm collections.

**Methods:**

In this study, a comprehensive panel of 210 rice varieties was phenotyped over ten days in controlled conditions, subjected to simulated drought stress using 20% PEG 6000 in petri dishes. Throughout the stress period, crucial traits such as germination percentage (GP), germination rate index (GRI), mean germination time (MGT), and seedling percentage (SP) were meticulously monitored.

**Results:**

The GWAS analysis uncovered a total of 38 QTLs associated with drought tolerance traits, including novel loci like *qMGT-5.2, qSP-3, qSP7.2*, and *qGP-5.2*. Additionally, RNA-seq analysis identified ten genes with significant expression differences under drought stress conditions. Notably, haplotype analysis pinpointed elite haplotypes in specific genes linked to heightened drought tolerance.

**Discussion:**

Overall, this study underscores the importance of GWAS in validating known genes while unearthing novel loci to enrich the genetic resources for enhancing drought tolerance in rice breeding programs.

## 1 Introduction

Drought stress poses a significant threat to global food security, particularly in regions where rice (Oryza sativa L.) serves as a staple crop. With over half of the world’s population relying on rice for sustenance (Islam et al., 2018), understanding and mitigating the impact of drought stress on rice production is paramount. Drought stress during critical growth stages such as germination and early seedling growth can severely compromise yield and quality, emphasizing the urgent need to elucidate the genetic mechanisms underlying drought tolerance at these stages.

While extensive literature has highlighted the vulnerability of rice to drought stress (Rasheed et al., 2020), gaps remain in our understanding of the genetic basis of drought tolerance, especially during germination and early seedling growth stages. Previous studies have identified key genes and quantitative trait loci (QTLs) associated with drought tolerance in rice, such as *OsSIZ1*, *OsNHX1*, *OsNAC10, OsIQM1*, *OsWRKY89, OsHsp17.4, OsHSP17.7, OsPP2C30*, and *OsPP48* (Ross et al., 2007; Sato and Yokoya, 2008; Jeong et al., 2010; Almeida et al., 2017; Mishra et al., 2017; Sarkar et al., 2019; Fan et al., 2021; Kim et al., 2022; Zheng et al., 2022). However, these studies often focus on a limited set of candidate genes, leaving a vast pool of genetic variation untapped. Moreover, the genetic architecture of drought tolerance traits during germination and early seedling growth stages may differ from those at later growth stages, necessitating focused investigation. Understanding the intricate mechanisms underlying drought tolerance during these early growth phases is crucial for developing drought-resilient rice varieties capable of withstanding environmental challenges.

In this context, Genome-wide association study (GWAS) emerges as a powerful tool for dissecting complex traits and identifying genetic variations associated with drought tolerance in rice. By leveraging the natural genetic variation present in diverse germplasm collections, GWAS enables the identification of candidate genes and genomic regions underlying target traits. Despite the progress made, there remains a critical gap in uncovering novel genes and genetic variations contributing to drought tolerance, especially during germination and early seedling growth stages.

Therefore, this study aims to bridge these research gaps by uncovering novel genes involved in drought stress response during the germination and early seedling growth stages in rice using GWAS. By analysing a diverse panel of rice germplasm collections, we seek to identify genetic variations associated with drought tolerance traits, including germination percentage, seedling percentage, germination rate index, and mean germination time under drought stress conditions. Through this comprehensive approach, we aim to elucidate the genetic mechanisms underlying drought tolerance at these critical stages, providing valuable insights into the genetic architecture of drought tolerance in rice. By addressing these research questions, we aim to contribute to the development of drought-resilient rice varieties, thereby enhancing global food security in the face of changing climatic conditions.

## 2 Materials and methods

### 2.1 Plant materials

A diverse set of 210 rice cultivars (**Supplementary Table S1**) was assembled for studying rice drought tolerance during germination. These cultivars were primarily collected from 15 provinces across China, alongside samples from the Philippines and Japan, representing a wide geographical range from 15° to 48° north latitude, including temperate, tropical, and subtropical regions. Among these, 56 were japonica rice and 152 were *Indica* rice. The collection of rice materials adhered to local regulations and were carried out over multiple generations in experimental fields at Jiangxi Agricultural University, Nanchang, Jiangxi Province, and Linwang, Hainan Province.

### 2.2 Evaluation of drought stress at germination stage

All seeds from each accession were dried at 45°C for 2 days to break seed dormancy. Subsequently, the seeds were surface sterilized with a 15% sodium hypochlorite solution for 15 minutes and rinsed three times with sterile distilled water before the germination experiment. The evaluation of seed germination under water stress conditions was conducted in Petri dishes by placing 30 seeds of each rice accession on a filter paper layer moistened with 25 mL of PEG 6000 solution with a concentration of 200 g/L, considered to be a severe concentration for germination according to Zhao et al. (2016), and distilled water was used as a control (Wu et al., 2021). Petri dishes were incubated in the dark at 28°C in a growth chamber. The evaluation followed a randomized complete block design (RCBD) with three repetitions. The seed germination period was 10 days, and the number of seeds from each accession that germinated was recorded daily. Seeds were considered germinated when the radicle protruded (1 mm) through the seed coat, and seedlings were considered established when the root length reached seed length (Peng et al., 2022). The PEG 6000 solution was changed every 2 days. All experiments were repeated three times. The germination energy (GE), germination rate index (GRI), seedling percentage (SP), germination percentage (GP), and days to 50% germination (R50) were calculated and subjected to a GWAS. The germination indices were calculated using the formulas described in Sagar et al. (2020); Li et al. (2022); Yuan et al. (2020).

1. GRI = Σ (Gt/t), where Gt is the number of seeds that germinated.
2. SP = (Total number of seedlings/Total number of seeds germinated) x 100%.
3. GP = (Total number of germinated seeds/Total number of seeds) x 100%.
4. MGT= Σ (Germination times/Number of germinated seeds).

### 2.3 Genome-wide association analysis

For the Genome-Wide Association Study (GWAS) investigating drought stress tolerance in rice during the germination stage, the initial quality control of genotype data was carried out using TASSEL 5.0 software (Bradbury et al., 2007). SNP sites with a deletion rate higher than 40% and a minimum allele frequency (MAF) below 5% were filtered out, resulting in a refined dataset of 2,858,438 SNP genotypes (Wei et al., 2021). The subsequent GWAS analysis was performed using the R package rMVP (Yin et al., 2023). Data formatting involved converting genotypic data into HapMap format and phenotypic data, specifying parameters for tab-delimited files, additive SNP effects, and generating a kinship matrix. The GWAS analysis followed a structured pipeline, utilizing three models: General Linear Model (GLM), Mixed Linear Model (MLM), and Fixed and Random Model Circulating Probability Unification (FarmCPU). These models incorporated different covariate structures, including five principal components (PCA), kinship matrix, and a combination of both. Visualization of results was achieved by creating Manhattan and QQ plots with customized settings for color, significance thresholds, and amplification. QTLs were identified where multiple SNPs exceeded the significance threshold of -log10 (P) ≥ 3.0. Detailed scripts and methodologies, including all parameter settings, can be accessed at https://github.com/xiaolei-lab/rMVP.

### 2.4 Candidate gene analyses

The LD blocks were utilized to identify candidate gene regions using R Studio software version 4.2.2. The SNPs with the most significant associations within a block were designated as the leading SNPs, and LD blocks containing significantly associated SNPs were defined as candidate genomic regions. Differentially expressed genes were identified using the Plant Public RNA-seq Database. The expression patterns of the differentially expressed genes were analyzed using publicly available microarray data from https://expression.ic4r.org/. Haplotype analyses were performed using RiceVarMap V2.0. Information on candidate genes was collected and classified by the NCBI, China Rice Data Centre, and the Rice Genome Annotation Project.

### 2.5 Quantitative real-time PCR analysis

Embryos of seeds germinated in water and in 20% PEG 6000 solutions for 2 days were collected for expression analysis. Total RNA was extracted using a MiniBEST Plant RNA Extraction kit (Takara, China). The corresponding gene sequences were obtained from the Rice Genome Annotation Project (http://rice.uga.edu/index.shtml). A total of 2.5 µl of RNA was reverse-transcribed into cDNA using the HiScript^®^ III RT SuperMix Kit. For quantitative real-time PCR, 1 µg of total RNA was reverse-transcribed into cDNA using the FastKing gDNA Dispelling RT SuperMix Kit (Tiangen; KR118–02). qRT-PCR analyses were conducted using the SuperReal PreMix Plus (SYBR Green) Kit (Tiangen; FP205–2) with three biological replicates. Throughout the study, OsActin was used as a reference gene. The relative expression level was calculated following the method described by (Livak and Schmittgen, 2001). The primers used for qRT-PCR can be found in **Supplementary Table S3**.

### 2.6 Statistical analysis and mapping

The mean values and standard errors of the phenotypic data were calculated using Microsoft Excel 2016. Correlation coefficients were calculated using R statistical software with the Corrplot (Wei and Simko, 2017) and metan packages (Olivoto and Lúcio, 2020). Bar graphs were created using Microsoft excel (2016) (Microsoft Corporation, 2016). R Studio 4.2.2 software (Team, 2022) was used to generate the LDheatmap with the LDheatmap package (Shin, 2006) and Manhattan plots with the RMVP package (Yin et al., 2023).

## 3 Results

### 3.1 Analysis of the phenotypes of the rice varieties

**Figure 1:**
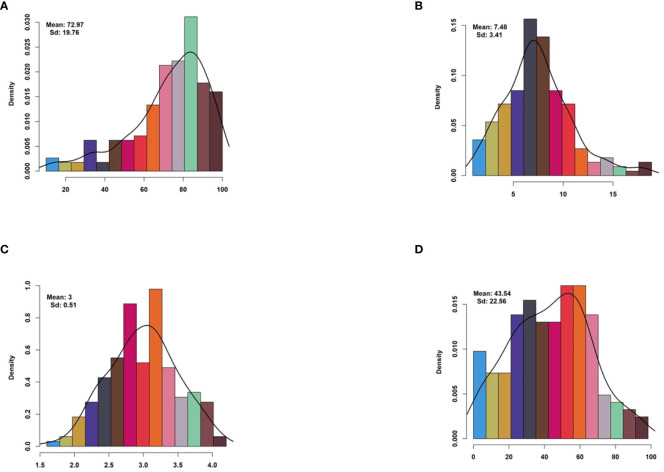
Histogram of drought-tolerance indices for drought-related traits. Histogram illustrating the distribution of drought-tolerance indices for four key drought-related traits: **(A)** Germination Percentage, **(B)** Germination Rate Index, **(C)** Mean Germination Time, and **(D)** Seedling Percentage. Each subplot represents the frequency distribution of the respective trait’s drought-tolerance index within the studied population. Drought-tolerance indices provide insights into the resilience of plant populations to drought stress, with higher indices indicating greater tolerance to adverse conditions.

The descriptive statistics offer valuable insights into the distribution and variability of germination and seedling-related traits among the rice varieties. The Germination Percentage (GP) had an average score of 72.97% (SD = 19.76), indicating significant variability in germination rates. Seedling Percentage (SP) showed a mean score of 43.54% (SD = 22.56), with a wide range from 0.0% to 100.0%. The germination rate index (GRI) had a mean value of 7.48 (SD = 3.41), suggesting moderate variability in germination rates. Mean Germination Time (MGT) had an average value of 3.00 (SD = 0.51), indicating consistent germination times across samples (**Figure 1** and **Table 1**). Skewness and kurtosis values varied across traits, reflecting different data distributions and shapes. The coefficients of variation ranged from moderate to high, indicating varying degrees of variability among the traits (**Table 1**). The diverse range and variability of these germination and seedling-related traits make the dataset suitable for Genome-Wide Association Studies (GWAS). GWAS could utilize this data to investigate potential genetic associations with these complex traits, revealing the genetic factors influencing germination and seedling establishment in plants.

**Table 1:** Statistical analysis of the drought-Tolerant index of measured traits in in rice population.

Trait	Mean	SD	Range	Skewness	Kurtosis	CV%
GP	72.97	19.76	10–100	-1.07	0.87	26.88
GRI	7.48	3.41	0.95–18.85	0.71	0.86	45.33
MGT	3.00	0.51	1.60–4.20	-0.03	-0.33	16.67
SP	43.54	22.56	0.0–100.0	0.14	-0.51	52.43

**Figure 2:**
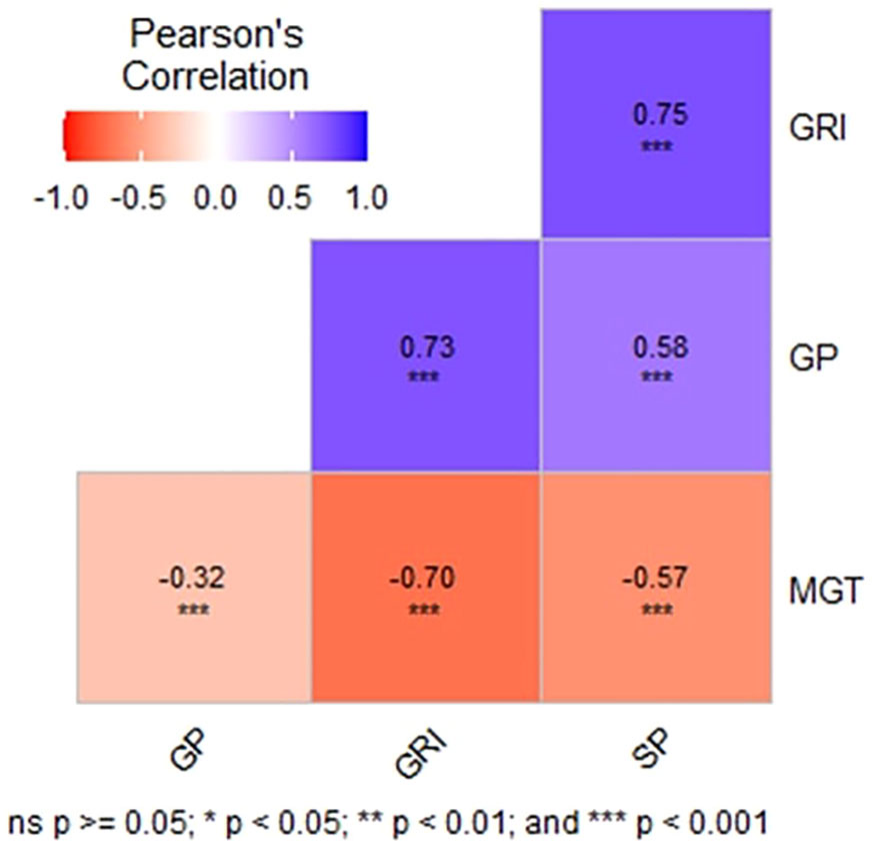
Correlation coefficients of tested variables. This figure displays the correlation coefficients between the tested variables, indicating the strength and direction of their relationships. Each coefficient represents the degree of linear association between pairs of variables, providing insights into their potential interdependencies. Positive coefficients denote a positive correlation, where increases in one variable are associated with increases in the other, while negative coefficients signify an inverse relationship. Correlation coefficients closer to 1 or -1 indicate stronger associations, whereas coefficients near 0 suggest weak or no correlation.

Pairwise Pearson’s correlation analysis (**Figure 2**) showed significant positive correlations among GP, GRI, and SP values under drought stress, suggesting shared genetic pathways. In contrast, MGT exhibited significant negative correlations with other parameters, indicating distinct genetic or physiological mechanisms governing this trait. These results highlight substantial genetic variation during the seedling stage under drought stress. The observed correlations and variability support the use of these results for Genome-Wide Association Studies (GWAS), which could further uncover the genetic basis of drought tolerance in rice and guide targeted breeding strategies during the germination stage.

### 3.2 Identification of QTLs for drought stress tolerance at germination stage

**Figure 3:**
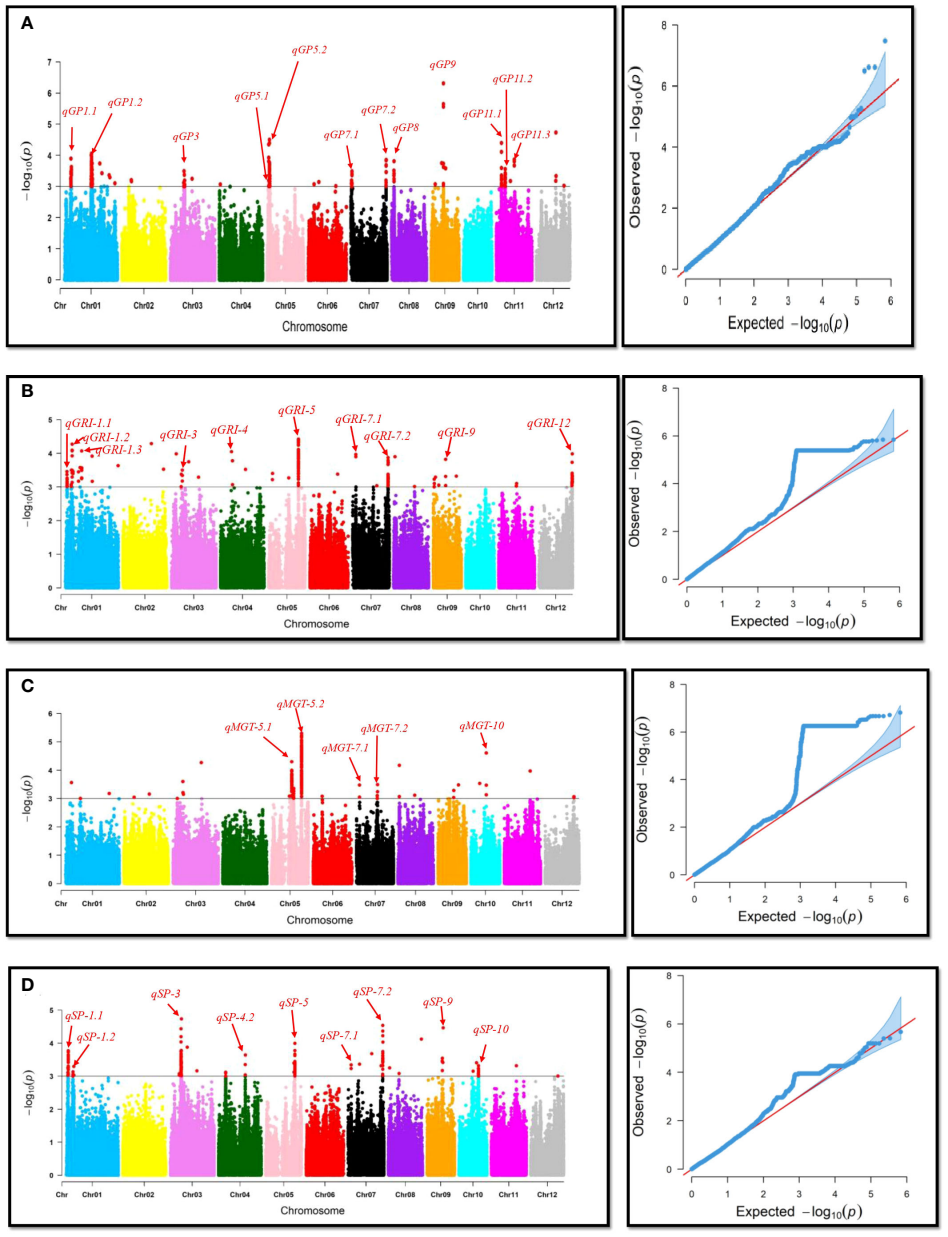
Manhattan and QQ-plots for genome-wide association of drought-related traits. This figure presents Manhattan and QQ-plots illustrating the results of genome-wide association studies (GWAS) for four drought-related traits: **(A)** Germination Percentage, **(B)** Germination Rate Index, **(C)** Mean Germination Time, and **(D)** Seedling Percentage. The Manhattan plots depict the strength and genomic locations of associations between genetic markers and the studied traits, with peaks representing significant marker-trait associations. The QQ-plots assess the deviation of observed p-values from the expected distribution under the null hypothesis of no association, aiding in the identification of potential genomic regions contributing to the variation in drought tolerance traits.

To investigate the genetic basis of natural variation in drought tolerance during the germination stage, a Genome-Wide Association Study (GWAS) was conducted, and Manhattan plots were created to identify significant associations of single-nucleotide polymorphisms (SNPs) with drought tolerance indexes (**Figure 3**). Quantitative Trait Loci (QTLs) were defined as regions where multiple SNPs (at least two) exceeded the significance threshold value of 1x10^-3^. A total of 38 QTLs for Germination Percentage (GP), Mean Germination Time (MGT), Germination Rate Index (GRI), and Seedling Percentage (SP) were identified, distributed across the genome. For example, from **Table 2**, *qSP-1.2* and *qGRI-1.1* were linked to Seedling Percentage (SP) and Germination Rate Index (GRI), respectively. These QTLs showed significant associations with a moderate proportion of phenotypic variance explained (PVE %). Additionally, *qGRI-1.2* had a higher PVE% and was associated with the gene *OsUBC34*, indicating its potential role in drought tolerance, as reported in previous studies (Zhiguo et al., 2015). Other noteworthy QTLs were identified on different chromosomes. Notably, *qSP-3* and *qGRI-3* on chromosome 3 displayed significant associations with SP and GRI, respectively, with substantial PVE%. These QTLs were linked to genes such as *OsHsp17.4*, *OsHSP17.7*, *OsPP2C30*, and *OsPP48*, as reported in previous studies (Sato and Yokoya, 2008; Kim et al., 2012; Zhang et al., 2017; Sarkar et al., 2019). Furthermore, QTLs associated with other traits like GP and MGT were also identified. For instance, *qGP-1.2* on chromosome 1 and *qGP-5.2* on chromosome 5 showed significant associations with GP and were associated with candidate genes *OsIQM1* (Fan et al., 2021) and *OsWRKY89* (Ross et al., 2007), respectively. However, *qMGT-5.2*, *qSP-3*, *qSP-7.2*, and *qGP-5.2* will be further investigated due to their significant associations and potential implications for drought tolerance and seed germination. These QTLs show promise for detailed exploration to uncover their underlying genetic mechanisms and their relevance to rice breeding for enhanced resilience to water deficit conditions.

**Table 2:** Summary of the significant SNPs detected by GWAS.

QTL ID.	Trait	Chr.	No. of Significant SNPs	Peak SNPs	PVE%	P-value	Previous QTLs/Genes	References
*qSP-1.2*	SP	1	4	1374740	2.14	0.00056		
*qGRI-1.1*	GRI	1	4	1375163	3.20	0.00035		
*qGRI-1.2*	GRI	1	20	1445678	5.85	0.00055	*OsUBC34*	(Zhiguo et al., 2015)
*qSP-1.3*	SP	1	5	1445678	3.08	0.00076		
*qSP-1.1*	SP	1	41	1651808	8.55	0.00017		
*qGP-1.1*	GP	1	105	5168653	9.05	0.000129		
*qGRI-1.3*	GRI	1	13	5557526	9.25	5.36-E05		
*qGP-1.2*	GP	1	297	22104507	11.25	8.80E-05	*OsIQM1*	(Fan et al., 2021)
*qGRI-1.4*	GRI	1	25	13418716	60.25	8.52E-05		
*qGRI-3*	GRI	3	3	8876470	20.88	0.00032		
*qSP-3*	SP	3	221	8882826	87.35	3.67E-05	*OsHsp17.4,OsHSP17.7, OsPP2C30; OsPP48*	(Sato and Yokoya, 2008; Kim et al., 2012; Zhang et al., 2017; Sarkar et al., 2019)
*qGP-3*	GP	3	8	11209936	8.31	0.00032		
*qSP-4.1*	SP	4	18	5848170	19.46	0.0010		
*qGRI-4*	GRI	4	2	9613048	14.31	0.000167		
*qSP-4.2*	SP	4	37	22201057	19.62	0.00093	*OsAL4*	(Yang et al., 2022)
*qGP-5.1*	GP	5	7	1240232	28.56	4.50E-05		
*qGP-5.2*	GP	5	178	1785033	36.48	3.16E-05	*OsWRKY89*	(Ross et al., 2007)
*qMGT-5.1*	MGT	5	75	16351872	16.57	5.05E-05		
*qMGT-5.2*	MGT	5	800	24189122	41.73	5.05E-06		
*qSP-5*	SP	5	612	24234457	11.71	0.0010		
*qGRI-5*	GRI	5	678	24239324	83.17	3.79E-05		
*qGP-7.1*	GP	7	33	809318	33.00	0.00033		
*qMGT-7.1*	MGT	7	21	2396459	4.69	0.00033		
*qGRI-7.1*	GRI	7	2	2511943	17.48	0.00010		
*qSP-7.1*	SP	7	2	2511943	39.73	0.00059		
*qMGT-7.2*	MGT	7	5	16912842	65.83	0.00032		
*qSP-7.2*	SP	7	96	28793074	21.98	2.91E-05	*OsWRKY47*	(Raineri et al., 2015)
*qGRI-7.2*	GRI	7	84	28793074	31.12	0.00013	*OsWRKY47*	(Raineri et al., 2015)
*qGP-7.2*	GP	7	20	28876288	19.31	0.00014		
*qGP-9*	GP	9	5	10175863	70.28	4.88E-07		
*qGRI-9*	GRI	9	4	10175863	29.54	0.00015		
*qSP-9*	SP	9	3	12768074	29.38	0.00029		
*qMGT-10*	MGT	10	3	12279419	65.91	2.48E-05		
*qSP-10*	SP	10	22	16235238	21.38	0.00049		
*qGP-11.1*	GP	11	101	4128457	44.80	4.04E-05		
*qGP-11.2*	GP	11	53	7276058	75.92	0.00028		
*qGP-11.3*	GP	11	3	14619221	0.11	0.00014		
*qGRI-12*	GRI	12	56	27186890	49.41	0.00010		

### 3.3 Candidate gene prediction and expression profiling

**Figure 4:**
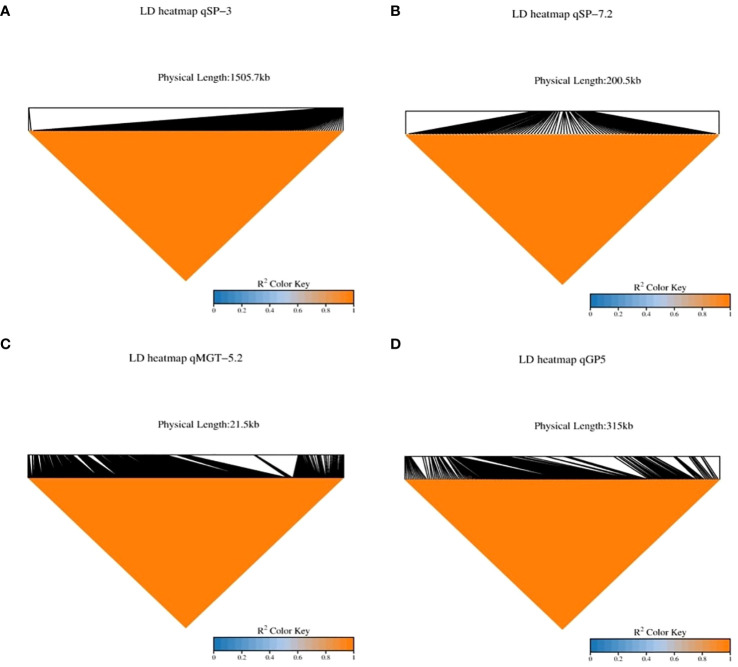
Linkage Disequilibrium (LD) heatmaps for strong Quantitative Trait Loci (QTLs). This figure showcases Linkage Disequilibrium (LD) heatmaps for several robust Quantitative Trait Loci (QTLs) associated with drought-related traits. The LD heatmaps provide insights into the degree of non-random association between genetic markers within genomic regions harboring significant QTLs. **(A)** qSP-3: LD heatmap illustrating the LD patterns surrounding the QTL qSP-3 associated with Seedling Percentage on chromosome 3. **(B)** qSP-7.2: LD heatmap depicting the LD structure around the QTL qSP-7.2 linked to Seedling Percentage on chromosome 7.2. **(C)** qMGT-5.2: LD heatmap displaying the LD relationships neighboring the QTL qMGT-5.2 correlated with Mean Germination Time on chromosome 5.2. **(D)** qGP-5: LD heatmap illustrating the LD profile adjacent to the QTL qGP-5 associated with Germination Percentage on chromosome 5.

In our study, we identified 38 QTLs associated with drought tolerance, focusing specifically on four: *qMGT-5.2, qSP-3, qSP7.2*, and *qGP-5.2*. LD decay analysis pinpointed significant regions: 1505.7 kb for *qSP-3*, 200.5 kb for *qSP-7.2*, 21.5 kb for *qMGT-5.2*, and 315 kb for *qGP-5.2* (**Figure 4**), suggesting these as promising candidates for enhancing drought tolerance during germination. To refine our selection of candidate genes, we excluded retrotransposons, transposons, hypothetical proteins, and unknown proteins, ultimately identifying 170 genes across these QTLs (**Supplementary Table S2**). Further screening based on gene annotation from the China Rice Data Centre (https://www.ricedata.cn/), accessed on 12 April 2024), and previous studies led us to pinpoint 21 genes potentially pivotal in drought stress during germination (**Table 3**).

**Table 3:** Candidate genes from the four QTLs.

No.	Gene ID.	Annotation
1.	LOC_Os07g48140	ATOFP17/OFP17, putative, expressed
2.	LOC_Os07g48200	B3 DNA binding domain containing protein, putative, expressed
3.	LOC_Os07g48229	Vacuolar-sorting receptor precursor, putative, expressed
4.	LOC_Os07g48300	Eukaryotic translation initiation factor 2 subunit beta, putative, expressed
5.	LOC_Os07g48320	HAD superfamily phosphatase, putative, expressed
6.	LOC_Os05g03810	Trehalose phosphatase, putative, expressed
7.	LOC_Os05g03840	Endoglucanase, putative, expressed
8.	LOC_Os05g03910	RNA polymerase II-associated protein 3, putative, expressed
9.	LOC_Os05g03920	TKL_IRAK_DUF26-lf.3 - DUF26 kinases have homology to DUF26 containing loci, expressed
10.	LOC_Os05g04020	Plant protein of unknown function domain containing protein, expressed
11.	LOC_Os05g04120	Ferroportin1 domain containing protein, expressed
12.	LOC_Os05g04160	Pentatricopeptide containing protein, putative, expressed
13.	LOC_Os05g04170	AMP-binding enzyme, putative, expressed
14.	LOC_Os05g04210	MYB family transcription factor, putative, expressed
15.	LOC_Os03g14990	Chorismate synthase 2, chloroplast precursor, putative, expressed
16.	LOC_Os03g15050	Phosphoenolpyruvate carboxykinase, putative, expressed
17.	LOC_Os03g15440	Basic helix-loop-helix, putative, expressed
18.	LOC_Os03g15860	Mitochondrial carrier protein, putative, expressed
19.	LOC_Os03g16050	Fructose-1,6-bisphosphatase, putative, expressed
20.	LOC_Os03g17180	ABC transporter, ATP-binding protein, putative, expressed
21.	LOC_Os03g17310	Calcium-transporting ATPase, endoplasmic reticulum-type, putative, expressed

To investigate gene expression changes in response to drought stress, we analysed RNA-Seq data from the Gene Expression Omnibus (GEO) database under accession number [GSE183241] (https://www.ncbi.nlm.nih.gov/geo/query/acc.cgi?acc=GSE183241). This dataset, from a single comprehensive study, included three biological replicates each for control (CK) and drought stress (DS) conditions. We selected these samples because they provided a controlled and consistent basis for analysing the impact of drought stress.

**Figure 5:**
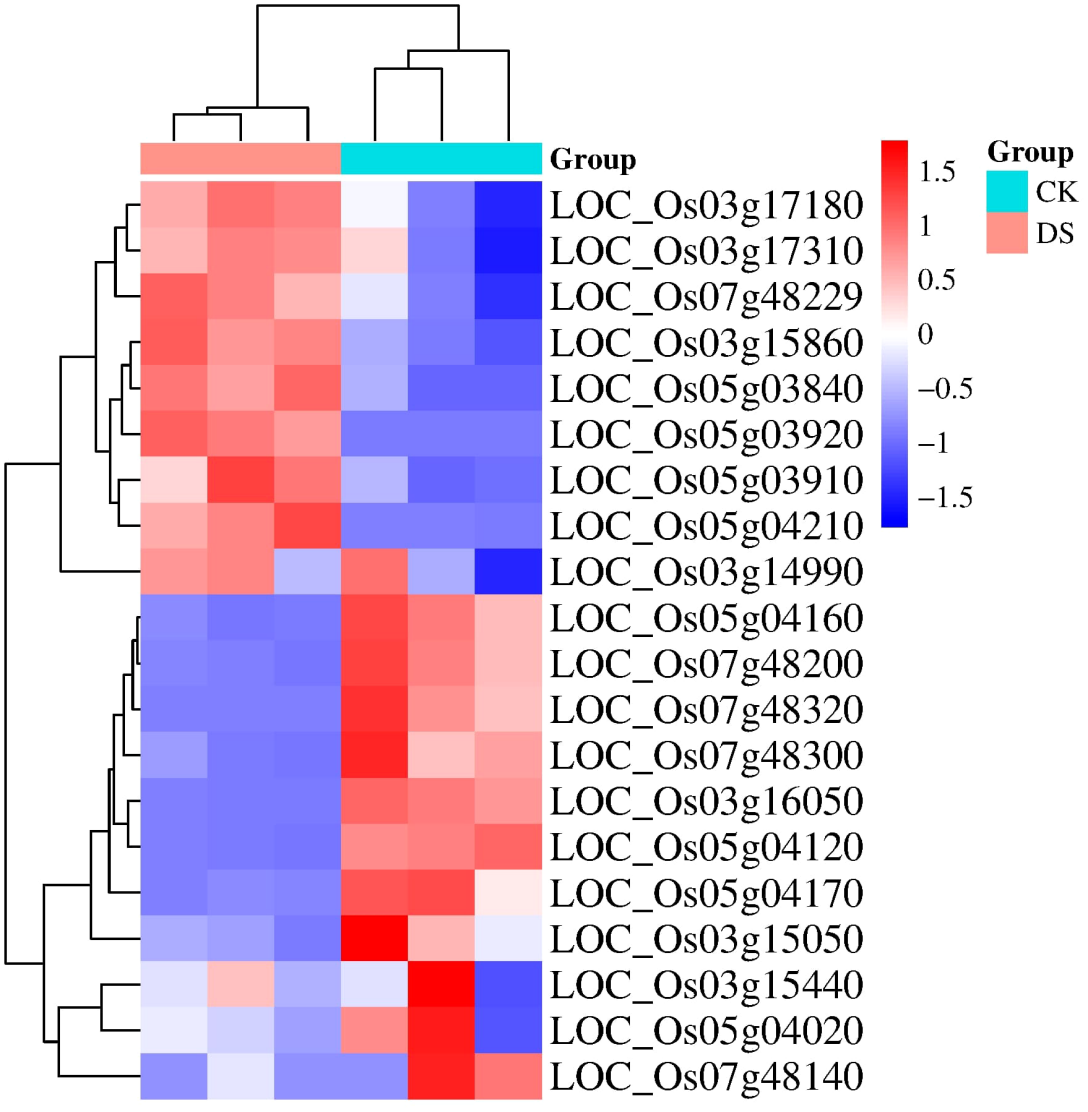
Shows a heatmap depicting the differential gene expression in rice seeds under drought stress. The RNA-Seq data from three biological replicates for both control (CK) and drought stress (DS) conditions were analyzed using DESeq2. The heatmap visualizes the expression patterns of genes that exhibit differential expression under drought stress.

We utilized the provided read counts to construct a heatmap (**Figure 5**) visualizing gene expression patterns under drought stress conditions. This analysis revealed 22 genes with distinct expression changes, with genes such as *LOC_Os03g17180, LOC_Os03g17310*, and *LOC_Os03g15860* upregulated under drought stress, while *LOC_Os03g14990* and *LOC_Os05g04160* were downregulated.

We selected the genes *LOC_Os05g04170, LOC_Os07g48200, LOC_Os03g14990, LOC_Os03g17310, LOC_Os03g17180, LOC_Os05g04210, LOC_Os05g03910, LOC_Os05g03840, LOC_Os07g48229*, and *LOC_Os03g15860* for further analysis due to their distinct expression patterns and functional annotations, which suggest significant roles in drought stress response mechanisms. Although some of these genes, such as *LOC_Os07g48200* and *LOC_Os05g04160*, were downregulated under drought stress, their functional roles are critical in the overall drought response mechanism, justifying their inclusion in further analysis.

### 3.4 Natural allelic variations of candidate genes contribute to drought tolerance at germination stage

**Figure 6:**
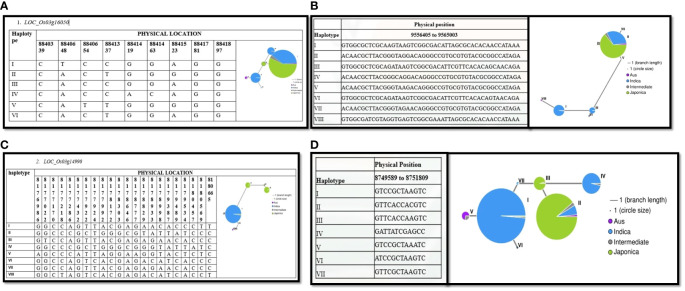
Haplotype analysis of candidate genes associated with drought tolerance. Presents haplotype analyses for four candidate genes associated with drought tolerance: **(A)** LOC_Os03g16050, **(B)** LOC_Os03g17180, **(C)** LOC_Os03g14990, and **(D)** LOC_Os03g15860. Each subfigure depicts the genetic diversity and allelic variation within the respective gene locus. The haplotype networks or allele frequency distributions provide insights into the allelic structures and genetic variants present within these genes. Such analyses are crucial for understanding the genetic basis of drought tolerance and identifying specific genetic variants or haplotypes associated with favorable phenotypic traits.

Our analysis revealed that most *Indica* rice varieties in our study outperformed japonica rice varieties. To understand the underlying reasons for these differences, we investigated the potential contributions of variations in candidate gene alleles. We conducted haplotype analyses using all non-synonymous single nucleotide polymorphisms (SNPs) within the open reading frames (ORFs) of the genes, employing RiceVarMap v2.0. Our analysis revealed distinct haplotypes for four genes between *Indica* and *Japonica* rice varieties (**Figure 6**). Collectively, these findings suggest that natural allelic variations in these four candidate genes are likely associated with the varying levels of drought tolerance observed among the 208 rice varieties.

**Figure 7:**
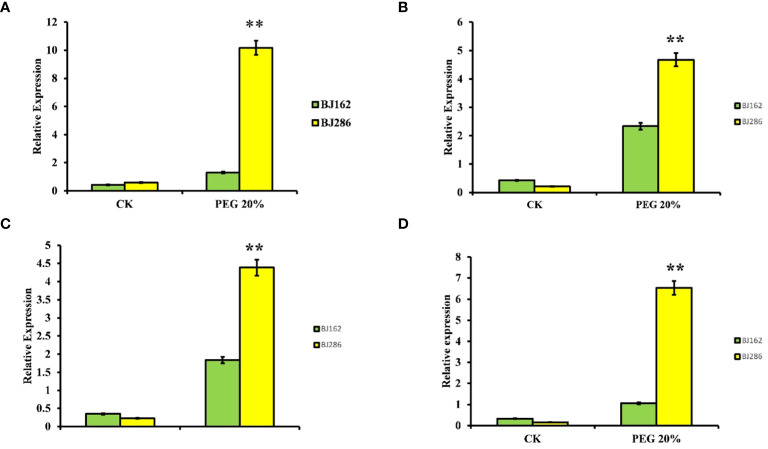
Expression Patterns of Candidate Genes Associated with Drought Tolerance. Illustrates the expression patterns of four candidate genes implicated in drought tolerance: LOC_Os03g14990 **(A)**, LOC_Os03g15860 **(B)**, LOC_Os03g16050 **(C)**, and LOC_Os03g17180 **(D)**. Each subplot displays the gene expression profiles under Control and drought conditions, providing insights into their transcriptional regulation in response to drought stress. Asterisks (**) denote statistically significant differences at p < 0.01.

To further confirm the response of the four genes to drought stress, we conducted quantitative real-time PCR (qRT-PCR). This analysis involved subjecting a drought-sensitive variety (BJ162) and a drought-tolerant variety (BJ286) to both control and drought stress conditions (PEG 6000 20%) (**Figure 7**). The results indicated differential expression of these four genes under drought stress (**Figure 7**). For *LOC_Os03g14990*, the expression was downregulated in the drought-sensitive variety and upregulated in the drought-tolerant variety under drought stress (**Figure 7A**). *LOC_Os03g15860* showed downregulation in the drought-sensitive variety and upregulation in the drought-tolerant variety under drought stress (**Figure 7B**). Similarly, *LOC_Os03g16050* exhibited downregulation in the drought-sensitive variety and upregulation in the drought-tolerant variety under drought stress (**Figure 7C**), while *LOC_Os03g17180* displayed downregulation in the drought-sensitive variety and upregulation in the drought-tolerant variety under drought stress (**Figure 7D**).

## 4 Discussion

The adoption of direct seeding in rice cultivation across various Asian countries has been propelled by its time-saving, labor-saving, and cost-effective advantages (Sha et al., 2019). However, maximizing the benefits of this approach and cultivating high-yield, high-quality rice varieties suitable for direct seeding necessitates robust germination abilities under diverse abiotic stresses and vigorous post-stress growth. Consequently, modern rice breeding has increasingly focused on developing varieties with high drought germination tolerance (Swain et al., 2017). Evaluating drought tolerance in seeds is crucial for breeding resilient crops, especially in the face of climate change-induced water scarcity (Kim and Lee, 2023). Recent studies underscore the significance of traits such as Germination Percentage (GP), Germination Rate Index (GRI), Mean Germination Time (MGT), and Seedling Percentage (SP) in assessing drought tolerance (Kim and Lee, 2023; Siddique et al., 2023; Akram et al., 2024; Benlioğlu et al., 2024; Maresca et al., 2024). GP reflects the proportion of seeds capable of germinating under water stress, providing insights into overall germination success (Khaeim et al., 2022), while GRI measures the speed of germination critical for adaptation to limited water availability (Amin et al., 2024). MGT signifies water use efficiency during germination, with shorter times indicating better drought adaptation (Gresta et al., 2018). SP evaluates the ability of seeds to germinate and grow into seedlings under conditions of limited water availability (Francki et al., 2021). These traits have played a pivotal role in Genome-Wide Association Studies (GWAS), facilitating the identification of genomic regions associated with drought tolerance and enabling marker-assisted selection and breeding of drought-resilient crop varieties. By integrating these traits into GWAS, researchers can decipher the genetic basis of drought tolerance, paving the way for developing climate-resilient crops crucial for global food security in a changing climate. The descriptive statistics further revealed significant variability in germination and seedling-related traits among rice varieties, highlighting the dataset’s suitability for GWAS to uncover genetic associations underlying these complex traits. Pearson’s correlation analysis unveiled significant positive correlations among GP, GRI, and SP values under drought stress, suggesting shared genetic pathways, while MGT exhibited significant negative correlations with other parameters, indicating distinct genetic or physiological mechanisms governing this trait. These results emphasize substantial genetic variation during the seedling stage, particularly under drought stress conditions, underscoring the potential utility of these findings for enhancing drought tolerance in rice through targeted breeding strategies.

The results of the Genome-Wide Association Study (GWAS) revealed a plethora of Quantitative Trait Loci (QTLs) associated with drought tolerance during the germination stage in rice (**Figure 3**). These findings have significant implications for rice breeding programs aiming to develop drought-resilient varieties. Among the 38 identified QTLs for traits including Germination Percentage (GP), Mean Germination Time (MGT), Germination Rate Index (GRI), and Seedling Percentage (SP), several exhibited substantial associations with phenotypic variance explained (PVE %) (**Table 2**). For instance, QTLs such as *qSP-1.2* and *qGRI-1.1* showed notable associations with moderate PVE% and were linked to Seedling Percentage (SP) and Germination Rate Index (GRI), respectively. These results align with previous studies suggesting the genetic basis of drought tolerance in rice (Islam et al., 2018; Rasheed et al., 2020; Hassan et al., 2023). Additionally, QTLs like *qSP-3* and *qGRI-3* on chromosome 3 displayed significant associations with SP and GRI, respectively, with substantial PVE%. These QTLs were associated with genes such as *OsHsp17.4, OsHSP17.7, OsPP2C30*, and *OsPP48*, consistent with prior research emphasizing the role of heat shock proteins and protein phosphatases in drought response mechanisms (Sato and Yokoya, 2008; Zhang et al., 2017; Sarkar et al., 2019; Kim et al., 2022). Furthermore, QTLs associated with other traits like GP and MGT were also identified. For instance, *qGP-1.2* on chromosome 1 and *qGP-5.2* on chromosome 5 exhibited significant associations with GP and were linked to candidate genes *OsIQM1* and *OsWRKY89*, respectively, corroborating previous findings highlighting the involvement of these genes in drought response pathways (Ross et al., 2007; Fan et al., 2021). However, further investigation of QTLs such as *qMGT-5.2*, *qSP-3, qSP-7.2*, and *qGP-5.2* is warranted due to their significant associations and potential implications for drought tolerance and seed germination. These results underscore the importance of genetic diversity and targeted breeding efforts in developing drought-resilient rice varieties essential for global food security in the face of climate change.

In this study, we identified a total of 170 genes across the four specific QTLs associated with drought tolerance during germination in rice. Among these genes, 21 were selected for further investigation based on promising annotations and prior studies. Notably, our analysis revealed ten genes (*LOC_Os05g04170, LOC_Os07g48200, LOC_Os03g14990, LOC_Os03g17310, LOC_Os03g17180, LOC_Os05g04210, LOC_Os05g03910, LOC_Os05g03840, LOC_Os07g48229*, and *LOC_Os03g15860*). Interestingly, the haplotype analysis suggested that the haplotypes of four candidate genes (*LOC_Os03g16050, LOC_Os03g14990, LOC_Os03g17180*, and *LOC_Os03g15860)* exhibited some indica–japonica specificity (**Figure 7**), which was consistent with our phenotypic results that the drought tolerance levels of indica and japonica rice were significantly different.

*LOC_Os03g16050* encodes fructose-1,6-bisphosphatase, putative protein. Fructose-1,6-bisphosphatase (FBPase) is a key enzyme in the Calvin cycle, essential for glucose production in plants (Zhu et al., 2023). Although not directly linked to drought stress tolerance during germination, its involvement in photosynthesis and energy metabolism suggests potential implications in stress responses. While drought stress may not directly target FBPase, its impact on water availability profoundly affects photosynthetic efficiency and subsequent metabolic processes. Evidence suggests that FBPase expression can be modulated under drought conditions (Xue et al., 2008; Wang et al., 2022) indicating its potential relevance in stress adaptation pathways. However, the precise contribution of FBPase to drought stress resilience during germination remains complex and context-dependent, influenced by factors such as plant species and environmental conditions. Further investigation is warranted to elucidate the specific mechanisms underlying FBPase involvement in drought stress responses during germination, thus contributing to a more comprehensive understanding of plant stress adaptation strategies.

*LOC_Os03g14990* encodes for chorismate synthase 2, chloroplast precursor protein. Chorismate synthase 2 (CS2) is a crucial enzyme involved in the biosynthesis of aromatic amino acids and secondary metabolites in plants, localized within the chloroplast (Zhong et al., 2020). While its specific role in drought tolerance during the germination stage in rice has not been directly studied, its localization in the chloroplast and involvement in metabolic pathways suggest potential relevance. The chloroplast plays a vital role in various physiological processes, including stress responses, and CS2’s participation in the shikimate pathway underscores its importance in plant metabolism (Chen et al., 2018). Furthermore, the shikimate pathway is essential for the synthesis of defense compounds and osmoprotectants, which are crucial for plants under stress conditions (Santos-Sánchez et al., 2019). Although empirical evidence linking CS2 to drought tolerance during germination in rice is lacking, research on related pathways and enzymes suggests that chloroplast metabolism influences stress responses at various developmental stages. Investigating the specific role of CS2 in drought stress responses during germination could provide valuable insights into its potential contribution to drought tolerance in rice.

*LOC_Os03g17180* encodes for ABC transporter, ATP-binding protein. The ATP-binding protein of ATP-binding cassette (ABC) transporters is implicated in plant responses to drought stress during the germination stage, although its precise involvement in rice remains to be fully elucidated. ABC transporters are known to participate in stress adaptation mechanisms, including drought responses (Dahuja et al., 2021). During germination, water availability is critical for seed imbibition and metabolic processes initiation, making this stage particularly sensitive to drought stress. Research suggests that ABC transporters, through their role in transporting phytohormones and secondary metabolites, may regulate water balance and stress signaling pathways (Do et al., 2021). The ATP-binding protein of ABC transporters provides the necessary energy for substrate transport, potentially influencing the transport of stress-related molecules essential for drought tolerance during germination. Thus, investigating the specific involvement of ATP-binding proteins of ABC transporters in drought stress responses during rice germination could offer insights into their contribution to drought tolerance mechanisms at this critical stage.

*LOC_Os03g15860* encodes for mitochondrial carrier protein. Mitochondrial carrier proteins are integral to cellular metabolism and have been implicated in plant responses to various environmental stresses, including drought. While their specific involvement during the germination stage in rice under drought conditions is not extensively characterized, studies in other plant species suggest their potential relevance. Mitochondria play essential roles in energy production and stress signalling, processes vital for germination under adverse conditions. Mitochondrial carrier proteins facilitate the transport of metabolites across the mitochondrial membrane, thereby influencing energy metabolism and stress responses (Ferramosca and Zara, 2021). During germination, maintenance of mitochondrial function is crucial for providing energy for cellular processes and adapting to stress. Research in Arabidopsis has shown that mitochondrial carrier proteins are responsive to drought stress, suggesting their potential involvement in stress tolerance mechanisms (Begcy et al., 2011). Investigating the specific role of mitochondrial carrier proteins in rice germination under drought stress could provide insights into their contribution to drought tolerance mechanisms and identify potential targets for crop improvement. These findings contribute to expanding our understanding of the genetic mechanisms underlying drought tolerance during germination in rice and provide valuable insights into potential targets for molecular breeding strategies.

## Data availability statement

The variation data reported in this paper have been deposited in the Genome Variation Map (GVM) in National Genomics Data Center, Beijing Institute of Genomics, Chinese Academy of Sciences and China National Center for Bioinformation, under accession number GVM000815, BioProject PRJCA028545. This data can be found here: https://bigd.big.ac.cn/gvm/getProjectDetail?Project=GVM000815.

## Author contributions

JB: Conceptualization, Funding acquisition, Supervision, Validation, Writing – review & editing. MN: Conceptualization, Data curation, Formal analysis, Methodology, Validation, Writing – original draft, Writing – review & editing. QZ: Investigation, Project administration, Validation, Writing – review & editing. XSL: Investigation, Validation, Writing – review & editing. XL: Investigation, Validation, Writing – review & editing. ZW: Investigation, Project administration, Writing – review & editing. LC: Investigation, Methodology, Project administration, Writing – review & editing. HH: Conceptualization, Supervision, Writing – review & editing.
