# Supplementary material for: Uncovering novel genes for drought stress in rice at germination stage using genome wide association study
Source: Front Plant Sci. 2024 Aug 1;15:1421267. doi: 10.3389/fpls.2024.1421267 (PMC11325455; doi:10.3389/fpls.2024.1421267)
Supplement: Supplementary file 2 [file Table_2.pdf]

Identified genes in the four QTLs

Number	Gene ID	Annotation
1.	LOC_Os03g14910	expressed protein
2.	LOC_Os03g14915	expressed protein
3.	LOC_Os03g14920	expressed protein
4.	LOC_Os03g14930	retrotransposon protein, putative, unclassified, expressed
5.	LOC_Os03g14940	retrotransposon protein, putative, unclassified
6.	LOC_Os03g14950	*OspPLAIIIα*
7.	LOC_Os03g14980	*OsTPR3; ASPR1*
8.	LOC_Os03g14990	chorismate synthase 2, chloroplast precursor, putative, expressed
9.	LOC_Os03g15000	Zinc finger, C3HC4 type domain containing protein, expressed
10.	LOC_Os03g15010	transposon protein, putative, unclassified, expressed
11.	LOC_Os03g15020	beta-galactosidase precursor, putative, expressed
12.	LOC_Os03g15033	expressed protein
13.	LOC_Os03g15040	FAR1 family protein, expressed
14.	LOC_Os03g15050	phosphoenolpyruvate carboxykinase, putative, expressed
15.	LOC_Os03g15070	expressed protein
16.	LOC_Os03g15080	expressed protein
17.	LOC_Os03g15090	expressed protein
18.	LOC_Os03g15100	expressed protein
19.	LOC_Os03g15110	expressed protein
20.	LOC_Os03g15120	imidazole glycerol phosphate synthase hisHF, chloroplast precursor, putative, expressed
21.	LOC_Os03g15130	expressed protein
22.	LOC_Os03g15140	retrotransposon protein, putative, unclassified, expressed
23.	LOC_Os03g15150	retrotransposon protein, putative, unclassified
24.	LOC_Os03g15160	retrotransposon protein, putative, Ty3-gypsy subclass, expressed
25.	LOC_Os03g15170	retrotransposon protein, putative, unclassified, expressed
26.	LOC_Os03g15180	PH domain containing protein, expressed
27.	LOC_Os03g15190	expressed protein
28.	LOC_Os03g15200	expressed protein
29.	LOC_Os03g15210	DUF292 domain containing protein, expressed
30.	LOC_Os03g15230	DUF292 domain containing protein, expressed
31.	LOC_Os03g15240	expressed protein
32.	LOC_Os03g15250	lectin-like receptor kinase, putative, expressed
33.	LOC_Os03g15260	expressed protein
34.	LOC_Os03g15270	gibberellin receptor GID1L2, putative, expressed
35.	LOC_Os03g15280	hypothetical protein
36.	LOC_Os03g15290	beclin-1, putative, expressed
37.	LOC_Os03g15300	hypothetical protein
38.	LOC_Os03g15310	transposon protein, putative, unclassified
39.	LOC_Os03g15320	glyoxal oxidase-related, putative, expressed
40.	LOC_Os03g15330	expressed protein
41.	LOC_Os03g15340	plastocyanin-like domain containing protein, putative, expressed

42.	LOC_Os03g15350	*OsHrd3*
43.	LOC_Os03g15360	nmrA-like family domain containing protein, expressed
44.	LOC_Os03g15370	*UbL402*
45.	LOC_Os03g15380	expressed protein
46.	LOC_Os03g15390	GA17271-PA, putative, expressed
47.	LOC_Os03g15400	retrotransposon protein, putative, unclassified, expressed
48.	LOC_Os03g15410	expressed protein
49.	LOC_Os03g15420	dynamin family protein, putative, expressed
50.	LOC_Os03g15430	Ser/Thr protein phosphatase family protein, putative, expressed
51.	LOC_Os03g15440	basic helix-loop-helix, putative, expressed
52.	LOC_Os03g15450	retrotransposon protein, putative, Ty3-gypsy subclass, expressed
53.	LOC_Os03g15460	expressed protein
54.	LOC_Os03g15470	expressed protein
55.	LOC_Os03g15480	heat shock protein DnaJ, putative, expressed
56.	LOC_Os03g15490	hypothetical protein
57.	LOC_Os03g15500	retrotransposon protein, putative, unclassified
58.	LOC_Os03g15510	transposon protein, putative, unclassified, expressed
59.	LOC_Os03g15520	transposon protein, putative, unclassified, expressed
60.	LOC_Os03g15530	expressed protein
61.	LOC_Os03g15540	HEAT repeat family protein, putative, expressed
62.	LOC_Os03g15560	wibg, putative, expressed
63.	LOC_Os03g15570	STE_MEKK_ste11_MAP3K.12 - STE kinases include homologs to sterile 7, sterile 11 and sterile 20 from yeast, expressed
64.	LOC_Os03g15580	AT hook motif family protein, expressed
65.	LOC_Os03g15590	eukaryotic translation initiation factor, putative, expressed
66.	LOC_Os03g15600	expressed protein
67.	LOC_Os03g15610	retrotransposon protein, putative, unclassified, expressed
68.	LOC_Os03g15620	retrotransposon protein, putative, unclassified, expressed
69.	LOC_Os03g15630	harpin-induced protein 1 domain containing protein, expressed
70.	LOC_Os03g15650	*GLUP6; GEF*
71.	LOC_Os03g15660	AP2 domain containing protein, expressed
72.	LOC_Os03g15670	hypothetical protein
73.	LOC_Os03g15680	nodulation-signaling pathway 2 protein, putative, expressed
74.	LOC_Os03g15690	phosphate carrier protein, mitochondrial precursor, putative, expressed
75.	LOC_Os03g15700	splicing factor 3A subunit 2, putative, expressed
76.	LOC_Os03g15710	*OsSTRL2*
77.	LOC_Os03g15720	expressed protein
78.	LOC_Os03g15730	S-RNase-binding protein, putative, expressed
79.	LOC_Os03g15740	uncharacterized TPR repeat-containing protein, putative, expressed
80.	LOC_Os03g15750	ASC1, putative, expressed
81.	LOC_Os03g15760	expressed protein
82.	LOC_Os03g15770	tyrosine protein kinase domain containing protein, putative, expressed
83.	LOC_Os03g15780	*OASA2*

84.	LOC_Os03g15790	ZOS3-08 - C2H2 zinc finger protein, expressed
85.	LOC_Os03g15800	B4-BTB2 - Bric-a-Brac, Tramtrack, Broad Complex BTB domain with B4 subfamily conserved sequence, expressed
86.	LOC_Os03g15810	AAA-type ATPase family protein, putative, expressed
87.	LOC_Os03g15820	expressed protein
88.	LOC_Os03g15830	transposon protein, putative, unclassified, expressed
89.	LOC_Os03g15840	*pls2*
90.	LOC_Os03g15850	josephin, putative, expressed
91.	LOC_Os03g15860	mitochondrial carrier protein, putative, expressed
92.	LOC_Os03g15870	ribosomal protein L4, putative, expressed
93.	LOC_Os03g15880	*OsCOI2*
94.	LOC_Os03g15890	RNA recognition motif containing protein, expressed
95.	LOC_Os03g15900	SH3 domain containing protein, expressed
96.	LOC_Os03g15910	membrane protein, putative, expressed
97.	LOC_Os03g15920	expressed protein
98.	LOC_Os03g15930	OTU-like cysteine protease family protein, putative, expressed
99.	LOC_Os03g15940	OsWLIM2 - LIM domain protein, putative actin-binding protein and transcription factor, expressed
100.	LOC_Os03g15950	enolase, putative, expressed
101.	LOC_Os03g15960	*OsHsp17.9A, affects seed germination*
102.	LOC_Os03g15970	transposon protein, putative, Mariner sub-class, expressed
103.	LOC_Os03g15990	EDM2, putative, expressed
104.	LOC_Os03g16000	src homology-3 domain protein 3, putative, expressed
105.	LOC_Os03g16010	BRASSINOSTEROID INSENSITIVE 1-associated receptor kinase 1 precursor, putative, expressed
106.	LOC_Os03g16020	*OsHsp17.4, affects seed germination and seedlings*
107.	LOC_Os03g16030	*OsHSP18.0-CI; OsMSR3*
108.	LOC_Os03g16040	*OsHSP17.7, drought tolerance*
109.	LOC_Os03g16050	fructose-1,6-bisphosphatase, putative, expressed
110.	LOC_Os03g16060	expressed protein
111.	LOC_Os03g16070	expressed protein
112.	LOC_Os03g16080	mitochondrial carrier protein, putative, expressed
113.	LOC_Os03g16090	*HDR3*
114.	LOC_Os03g16110	*OsPP1a*
115.	LOC_Os03g16120	myosin heavy chain-related, putative, expressed
116.	LOC_Os03g16130	CAMK_CAMK_like_ULKh_APGy.2 - CAMK includes calcium/calmodulin depedent protein kinases, expressed
117.	LOC_Os03g16140	digalactosyldiacylglycerol synthase, chloroplast precursor, putative, expressed
118.	LOC_Os03g16150	*OsVTC1-3; OsMPG3*
119.	LOC_Os03g16160	hypothetical protein
120.	LOC_Os03g16170	*OsPP2C30; OsPP48,drought tolerance*
121.	LOC_Os03g16200	hypothetical protein
122.	LOC_Os03g16210	tropinone reductase, putative, expressed
123.	LOC_Os03g16220	oxidoreductase, short chain dehydrogenase/reductase family domain containing protein, expressed
124.	LOC_Os03g16230	oxidoreductase, short chain dehydrogenase/reductase family protein, putative, expressed
125.	LOC_Os03g16250	retrotransposon protein, putative, unclassified

126.	LOC_Os03g16260	protein kinase, putative, expressed
127.	LOC_Os03g16270	expressed protein
128.	LOC_Os03g16280	retrotransposon protein, putative, unclassified, expressed
129.	LOC_Os03g16290	fringe-related protein, putative, expressed
130.	LOC_Os03g16300	transposon protein, putative, unclassified, expressed
131.	LOC_Os03g16310	retrotransposon protein, putative, LINE subclass, expressed
132.	LOC_Os03g16320	expressed protein
133.	LOC_Os03g16334	fringe-related protein, putative, expressed
134.	LOC_Os03g16350	DNA binding protein, putative, expressed
135.	LOC_Os03g16369	retrotransposon protein, putative, unclassified, expressed
136.	LOC_Os03g16390	avr9/Cf-9 rapidly elicited protein, putative, expressed
137.	LOC_Os03g16400	expressed protein
138.	LOC_Os03g16410	expressed protein
139.	LOC_Os03g16420	retrotransposon protein, putative, unclassified, expressed
140.	LOC_Os03g16430	RNA polymerase sigma factor, putative, expressed
141.	LOC_Os03g16440	outer membrane protein, OMP85 family, putative, expressed
142.	LOC_Os03g16450	pentatricopeptide, putative, expressed
143.	LOC_Os03g16460	expressed protein
144.	LOC_Os03g16470	expressed protein
145.	LOC_Os03g16480	zinc finger family protein, putative, expressed
146.	LOC_Os03g16490	hypothetical protein
147.	LOC_Os03g16500	aspartic proteinase nepenthesin precursor, putative, expressed
148.	LOC_Os03g16530	retrotransposon protein, putative, unclassified, expressed
149.	LOC_Os03g16540	transposon protein, putative, unclassified, expressed
150.	LOC_Os03g16550	expressed protein
151.	LOC_Os03g16560	expressed protein
152.	LOC_Os03g16570	*OsSDIR1, drought tolerance*
153.	LOC_Os03g16580	expressed protein
154.	LOC_Os03g16600	expressed protein
155.	LOC_Os03g16610	laccase precursor protein, putative, expressed
156.	LOC_Os03g16620	transposon protein, putative, unclassified, expressed
157.	LOC_Os03g16630	retrotransposon protein, putative, Ty1-copia subclass, expressed
158.	LOC_Os03g16640	retrotransposon protein, putative, Ty3-gypsy subclass
159.	LOC_Os03g16650	retrotransposon protein, putative, Ty1-copia subclass
160.	LOC_Os03g16660	expressed protein
161.	LOC_Os03g16670	haloacid dehalogenase-like hydrolase family protein, putative, expressed
162.	LOC_Os03g16680	hypothetical protein
163.	LOC_Os03g16690	oxysterol-binding protein, putative, expressed
164.	LOC_Os03g16700	initiator-binding protein, putative, expressed
165.	LOC_Os03g16709	expressed protein
166.	LOC_Os03g16718	expressed protein
167.	LOC_Os03g16730	expressed protein
168.	LOC_Os03g16740	*OsRLCK107*
169.	LOC_Os03g16750	retrotransposon protein, putative, Ty3-gypsy subclass, expressed
170.	LOC_Os03g16760	protein phosphatase 2C, putative, expressed

171.	LOC_Os03g16770	hypothetical protein
172.	LOC_Os03g16780	ankyrin repeat family protein, putative, expressed
173.	LOC_Os03g16790	DHHC zinc finger domain containing protein, expressed
174.	LOC_Os03g16800	clathrin assembly protein, putative, expressed
175.	LOC_Os03g16824	spotted leaf 11, putative, expressed
176.	LOC_Os03g16840	expressed protein
177.	LOC_Os03g16850	*OsDof-14; OsDof12*
178.	LOC_Os03g16860	*OsHSP71.1, drought tolerance*
179.	LOC_Os03g16870	expressed protein
180.	LOC_Os03g16874	expressed protein
181.	LOC_Os03g16880	DnaK family protein, putative, expressed
182.	LOC_Os03g16890	N-acetylglucosaminyltransferase, putative, expressed
183.	LOC_Os03g16900	*OsGDI3*
184.	LOC_Os03g16910	SLT1 protein, putative, expressed
185.	LOC_Os03g16920	DnaK family protein, putative, expressed
186.	LOC_Os03g16940	*OsGLYI3, seed salt stress tolerance*
187.	LOC_Os03g16950	cysteine-rich repeat secretory protein 55 precursor, putative, expressed
188.	LOC_Os03g16970	expressed protein
189.	LOC_Os03g16980	*BC25; OsUXS3*
190.	LOC_Os03g16990	expressed protein
191.	LOC_Os03g17000	NAD dependent epimerase/dehydratase family domain containing protein, expressed
192.	LOC_Os03g17010	RNA recognition motif containing protein, putative, expressed
193.	LOC_Os03g17020	GTPase-activating protein, putative, expressed
194.	LOC_Os03g17030	polyadenylate-binding protein, putative, expressed
195.	LOC_Os03g17040	expressed protein
196.	LOC_Os03g17050	expressed protein
197.	LOC_Os03g17060	RNA recognition motif containing protein, putative, expressed
198.	LOC_Os03g17070	ATP synthase B chain, chloroplast precursor, putative, expressed
199.	LOC_Os03g17084	Core histone H2A/H2B/H3/H4 domain containing protein, putative, expressed
200.	LOC_Os03g17100	Core histone H2A/H2B/H3/H4 domain containing protein, putative, expressed
201.	LOC_Os03g17110	transposon protein, putative, CACTA, En/Spm sub-class, expressed
202.	LOC_Os03g17120	arginine biosynthesis bifunctional protein argJ 1, putative, expressed
203.	LOC_Os03g17130	bHelix-loop-helix transcription factor, putative, expressed
204.	LOC_Os03g17140	expressed protein
205.	LOC_Os03g17150	ZOS3-09 - C2H2 zinc finger protein, expressed
206.	LOC_Os03g17164	kinesin-related protein, putative, expressed
207.	LOC_Os03g17170	zinc finger, C3HC4 type domain containing protein, expressed
208.	LOC_Os03g17174	PsbP, putative, expressed
209.	LOC_Os03g17180	ABC transporter, ATP-binding protein, putative, expressed
210.	LOC_Os03g17200	plant-specific domain TIGR01589 family protein, expressed
211.	LOC_Os03g17210	expressed protein
212.	LOC_Os03g17220	dirigent, putative, expressed

213.	LOC_Os03g17230	*OsUXS2*
214.	LOC_Os03g17240	ankyrin repeat-containing protein, putative, expressed
215.	LOC_Os03g17250	ankyrin repeat-containing protein, putative, expressed
216.	LOC_Os03g17260	expressed protein
217.	LOC_Os03g17270	expressed protein
218.	LOC_Os03g17280	expressed protein
219.	LOC_Os03g17300	TKL_IRAK_CrRLK1L-1.7 - The CrRLK1L-1 subfamily has homology to the CrRLK1L homolog, expressed
220.	LOC_Os03g17310	calcium-transporting ATPase, endoplasmic reticulum-type, putative, expressed
221.	LOC_Os07g48070	conserved hypothetical protein
222.	LOC_Os07g48080	expressed protein
223.	LOC_Os07g48100	*OsCIPK2*
224.	LOC_Os07g48110	expressed protein
225.	LOC_Os07g48120	expressed protein
226.	LOC_Os07g48130	*OsHAK9*
227.	LOC_Os07g48140	ATOFP17/OFP17, putative, expressed
228.	LOC_Os07g48150	DUF623 domain containing protein, expressed
229.	LOC_Os07g48160	alpha-galactosidase precursor, putative, expressed
230.	LOC_Os07g48170	nucleotidyltransferase, putative, expressed
231.	LOC_Os07g48180	*OsbZIP61*
232.	LOC_Os07g48190	expressed protein
233.	LOC_Os07g48200	B3 DNA binding domain containing protein, putative, expressed
234.	LOC_Os07g48210	retrotransposon protein, putative, unclassified
235.	LOC_Os07g48229	vacuolar-sorting receptor precursor, putative, expressed
236.	LOC_Os07g48244	ubiquinol-cytochrome c reductase complex 6.7 kDa protein, putative, expressed
237.	LOC_Os07g48260	*OsWRKY47 (draught stress)*
238.	LOC_Os07g48270	*CHR739*
239.	LOC_Os07g48280	expressed protein
240.	LOC_Os07g48290	AGC_AGC_other_RS6K_like.2 - ACG kinases include homologs to PKA, PKG and PKC, expressed
241.	LOC_Os07g48300	eukaryotic translation initiation factor 2 subunit beta, putative, expressed
242.	LOC_Os07g48310	inactive receptor kinase At2g26730 precursor, putative, expressed
243.	LOC_Os07g48320	HAD superfamily phosphatase, putative, expressed
244.	LOC_Os07g48330	*CYP714B1*
245.	LOC_Os07g48340	*OsCML24*
246.	LOC_Os05g41280	*OsbZIP43*
247.	LOC_Os05g41290	disease resistance RPP13-like protein 1, putative, expressed
248.	LOC_Os05g41300	expressed protein
249.	LOC_Os05g41310	disease resistance protein RGA2, putative, expressed
250.	LOC_Os07g48140	ATOFP17/OFP17, putative, expressed
251.	LOC_Os07g48200	B3 DNA binding domain containing protein, putative, expressed
252.	LOC_Os07g48229	Vacuolar-sorting receptor precursor, putative, expressed

253.	LOC_Os07g48300	Eukaryotic translation initiation factor 2 subunit beta, putative, expressed
254.	LOC_Os07g48320	HAD superfamily phosphatase, putative, expressed
255.	LOC_Os05g03810	Trehalose phosphatase, putative, expressed
256.	LOC_Os05g03840	Endoglucanase, putative, expressed
257.	LOC_Os05g03910	RNA polymerase II-associated protein 3, putative, expressed
258.	LOC_Os05g03920	TKL_IRAK_DUF26-lf.3 - DUF26 kinases have homology to DUF26 containing loci, expressed
259.	LOC_Os05g04020	Plant protein of unknown function domain containing protein, expressed
260.	LOC_Os05g04120	Ferroportin1 domain containing protein, expressed
261.	LOC_Os05g04160	Pentatricopeptide containing protein, putative, expressed
262.	LOC_Os05g04170	AMP-binding enzyme, putative, expressed
263.	LOC_Os05g04210	MYB family transcription factor, putative, expressed
